# Unkeito promotes follicle development by restoring reduced follicle-stimulating hormone responsiveness in rats with polycystic ovary syndrome

**DOI:** 10.3389/fendo.2023.1228088

**Published:** 2023-09-18

**Authors:** Sayako Yoshita, Satoko Osuka, Tomofumi Shimizu, Naoki Fujitsuka, Chinami Matsumoto, Bayasula, Natsuki Miyake, Ayako Muraoka, Natsuki Nakanishi, Tomoko Nakamura, Maki Goto, Hiroaki Kajiyama

**Affiliations:** ^1^ Department of Obstetrics and Gynecology, Nagoya University Graduate School of Medicine, Showa-ku, Nagoya, Aichi, Japan; ^2^ Tsumura Kampo Research Laboratories, Kampo Research & Development Division, Tsumura & Co., Yoshiwara, Ami-machi, Inashiki-gun, Ibaraki, Japan; ^3^ Bell Research Center for Reproductive Health and Cancer, Nagoya University Graduate School of Medicine, Tsurumai-cho, Showa-ku, Japan

**Keywords:** unkeito, wen-jing-tang, kampo medicine, polycystic ovary syndrome model, follicle-stimulating hormone receptor

## Abstract

**Background:**

Polycystic ovary syndrome (PCOS) is a common disorder resulting in irregular menstruation and infertility due to improper follicular development and ovulation. PCOS pathogenesis is mediated by downregulated follicle-stimulating hormone receptor (FSHR) expression in granulosa cells (GCs); however, the underlying mechanism remains elusive. Unkeito (UKT) is a traditional Japanese medicine used to treat irregular menstruation in patients with PCOS. In this study, we aimed to confirm the effectiveness of UKT in PCOS by focusing on follicle-stimulating hormone (FSH) responsiveness.

**Methods:**

A rat model of PCOS was generated by prenatal treatment with 5α-dihydrotestosterone. Female offspring (3-week-old) rats were fed a UKT mixed diet or a normal diet daily. To compare the PCOS phenotype in rats, the estrous cycle, hormone profiles, and ovarian morphology were evaluated. To further examine the role of FSH, molecular, genetic, and immunohistological analyses were performed using ovarian tissues and primary cultured GCs from normal and PCOS model rats.

**Results:**

UKT increased the number of antral and preovulatory follicles and restored the irregular estrous cycle in PCOS rats. The gene expression levels of FSHR and bone morphogenetic protein (BMP)-2 and BMP-6 were significantly decreased in the ovarian GCs of PCOS rats compared to those in normal rats. UKT treatment increased FSHR staining in the small antral follicles and upregulated *Fshr* and *Bmps* expression in the ovary and GCs of PCOS rats. There was no change in serum gonadotropin levels. In primary cultured GCs stimulated by FSH, UKT enhanced estradiol production, accompanied by increased intracellular cyclic adenosine monophosphate levels, and upregulated the expression of genes encoding the enzymes involved in local estradiol synthesis, namely *Cyp19a1* and *Hsd17b*. Furthermore, UKT elevated the expression of *Star* and *Cyp11a1*, involved in progesterone production in cultured GCs in the presence of FSH.

**Conclusions:**

UKT stimulates ovarian follicle development by potentiating FSH responsiveness by upregulating BMP-2 and BMP-6 expression, resulting in the recovery of estrous cycle abnormalities in PCOS rats. Restoring the FSHR dysfunction in the small antral follicles may alleviate the PCOS phenotype.

## 1 Introduction

Polycystic ovary syndrome (PCOS) is a common disorder characterized by polycystic ovaries, ovulation disorders, menstrual abnormalities, endocrinopathy, and several other features, including psychiatric and metabolic disorders, accounting for 8–13% of infertility cases in women (1). Despite its high prevalence, the pathogenesis of PCOS remains unclear, and fundamental treatments have not yet been developed. Fertility drugs used for ovarian stimulation have a narrow therapeutic range owing to the risk of ovarian hyperstimulation syndrome, making it difficult to treat PCOS (1).

The traditional Japanese medicine, unkeito (UKT), also known as wen-jing-tang in China, is a powdered extract comprising 12 medicinal herbs. This medicine is commonly used to treat menstrual irregularities in Asian countries and its clinical prescription in Japan has been approved by the Japanese Ministry of Health, Labour and Welfare. Several studies have reported the effects of UKT on reproductive function (2–7). Ushiroyama et al. (2, 3) reported that UKT induces substantial development of dominant follicles, improves the menstrual cycle in patients with PCOS, and prolongs the luteal phase in patients with luteal phase defects. Similarly, UKT is effective in treating ovulation disorders and steroid hormone deficiency (4, 5). *In vitro* studies have shown that UKT stimulates steroidogenesis and cytokine secretion in human granulosa cells (GCs) and rat ovarian cells (6, 7). However, the molecular mechanisms underlying the effects of UKT on patients with PCOS are poorly understood.

Gonadotropins, steroid hormones, cytokines, and other autocrine factors play a critical role in controlling follicle development and ovulation. High levels of luteinizing hormone (LH) and relatively low levels of follicle-stimulating hormone (FSH) are observed in patients with PCOS, which causes premature growth arrest in small follicles (8). Additionally, FSH receptor (FSHR) polymorphisms might affect the clinical phenotype of PCOS (9). A recent report (10) demonstrated that FSHR expression is downregulated in GCs from patients with PCOS compared to those from women with regular cycles. Noroozzadeh et al. (11) also reported the downregulation of FSHR expression in GCs of prenatal testosterone-treated PCOS rats. Therefore, reduced FSH responsiveness may partially contribute to abnormal follicular development in patients with PCOS. Thus, we hypothesized that UKT stimulates FSH activity in the ovary, resulting in the restoration of follicular development in cases of PCOS.

We previously demonstrated that prenatally 5α-dihydrotestosterone (DHT)-treated rats exhibited irregular estrous cycles with a decreased number of ovarian follicles and a PCOS-like phenotype (12). In this study, we aimed to confirm the effectiveness of UKT in treating disrupted estrous cycle and follicle development in a rat model of PCOS. Furthermore, we explored the mechanism of action of UKT by focusing on FSH responsiveness in GCs from animals with PCOS.

## 2 Materials and methods

### 2.1 Drugs and reagents

Powdered UKT extracts (lot nos. 362187700 and 382037400) were manufactured and supplied by Tsumura & Co. (Tokyo, Japan). UKT comprises 12 crude drugs in the following composition: four parts ophiopogon root (root of *Ophiopogon japonicus* Ker-Gawler, Liliaceae), four parts pinellia tuber (tuber of *Pinellia ternata* Breitenbach, Araceae), three parts Japanese angelica root (root of *Angelica acutiloba* Kitagawa, Umbelliferae), two parts glycyrrhiza (root of *Glycyrrhiza uralensis* Fischer, Leguminosae), two parts cinnamon bark (bark of *Cinnamomum cassia* J. Presl, Lauraceae), two parts peony root (root of *Paeonia lactiflora* Pallas, Paeoniaceae), two parts cnidium rhizome (rhizome of *Cnidium officinale* Makino, Umbelliferae), two parts ginseng (root of *Panax ginseng* C. A. Meyer, Araliaceae), two parts moutan bark (root bark of *Paeonia suffruticosa* Andrews, Paeoniaceae), one part euodia fruit (fruit of *Euodia ruticarpa* Hooker filius et Thomson, Rutaceae), one part ginger (rhizoma of *Zingiber officinale* Roscoe, Zingiberaceae), and two parts gelatin (i.e., alternative to Asini Corii Collas). A mixture of the constituent crude drugs was extracted using purified hot water for 1 h and the dried extract powder was obtained by spray drying. DHT was purchased from Tokyo Chemical Industry (Tokyo, Japan).

### 2.2 Animals

Female Wistar rats (pregnant [15 rats] or 3-week-old [35 rats]) were purchased from Charles River Laboratories (Yokohama, Japan). All animals were housed in cages and lighting (12-h light/dark cycle) and temperature (20–25°C) were controlled. They were provided ad libitum access to water and an animal diet (CE-2; CLEA Japan, Tokyo, Japan, or MF; Oriental Yeast, Tokyo, Japan) during acclimatization.

Pregnant rats (gestational day 16) were subcutaneously administered 3 mg DHT for 4 days. Their female offspring were used as a prenatally DHT-induced rat model of PCOS, as reported previously (12, 13).

CE-2 diet (normal diet) and CE-2 diet containing 3% UKT (UKT diet) were prepared by CLEA. After weaning on postnatal day (PND) 21, female offspring were randomly divided into two groups of DHT-induced PCOS rats fed CE-2 diet (DHT group) and DHT-induced PCOS rats fed UKT diet (DHT+UKT group).

All protocols were approved by the Animal Experiment Committee of Nagoya University Graduate School of Medicine (approval no. M210146) and Tsumura & Co. (approval nos. 19-052, 19-059, and 20-035). The care and use of the rats followed the guidelines of the Animal Experiment Committee of the Nagoya University Graduate School of Medicine and Tsumura & Co., as well as other relevant guidelines and regulations.

### 2.3 Assessment of estrous cyclicity

Vaginal smears of all rats were examined daily from PND 56 to PND 67 to assess the estrous cycle. Vaginal smear cells from each animal were viewed and classified as diestrus (D), proestrus (P), estrus (E), or metestrus (M) based on their shape and density, as previously described (14). The length of each estrous cycle was determined as the number of consecutive days from the one-cornified cell phase to the day before the next cornified cell phase. According to the classification detailed by Ishii et al. (15), we classified the estrous cycles as follows: normal estrous cycle (4-day or 5-day cycle, i.e., PEXX, PPXX, EEXX, or PEEXX); irregular estrous cycles, including shortened estrous cycle (3-day cycle, i.e., PXX or EXX); persistent estrous cycle (at least 4 days of sequential P or E), and persistent diestrus (at least 3 days of sequential M or D). XX indicates DD, DM, MD, or MM. Sequential vaginal smear scores were defined as a combination of normal 4- and 5-day cycles, shortened 3-day cycles, persistent estrus, or persistent diestrus (0, at least two normal cycles; 1, at least one normal cycle and at least one shortened cycle; 2, at least two shortened cycles; 3, at least one normal or shortened cycle and persistent diestrus; 4, no cycles with at least one persistent estrus, and 5, no cycles with at least one persistent diestrus).

### 2.4 Collection of blood and ovary samples

The rats were euthanized under isoflurane anesthesia during diestrus or metestrus on PND 70–90. Blood samples were collected immediately from the heart by needle aspiration. The serum was isolated by centrifugation at 3000 × *g* for 10 min and stored at -80°C until measurement. One ovary was removed from each animal, fixed in 10% formalin, embedded in paraffin, and stored at -80°C.

### 2.5 Hormone level measurements *In vivo*

LH and FSH concentrations were determined using rat LH and FSH enzyme-linked immunosorbent assay (ELISA) kits (FUJIFILM Wako Shibayagi Corporation, Gunma, Japan), respectively. Serum concentrations of estradiol (E2), progesterone (P4), and testosterone (T) were measured using an electrochemiluminescence immunoassay (SRL, Tokyo, Japan).

### 2.6 Analysis of ovarian morphology

Ovarian tissues were fixed in 10% neutral-buffered formalin and the paraffin-embedded tissue sections (6-µm) were stained with hematoxylin and eosin. Ovarian follicles were counted in the DHT (n = 6) and DHT+UKT (n = 5) groups. Follicles were classified as follows: preantral follicles (oocyte with two to five layers of cuboidal GCs), small antral follicles (oocyte surrounded by more than five layers of GCs, one or two small areas of follicular fluid, or both), large antral follicles (containing a single large antral cavity), preovulatory follicles (possessing a single large antrum and an oocyte surrounded by cumulus cells at the end of a stalk of mural GCs), and atretic cyst-like follicles (large fluid-filled cyst with an attenuated GC layer and dispersed theca cell layer) (16, 17). Follicle distribution was recorded as the follicle type for each ovary. Images of the entire ovary were examined side-by-side to prevent over-counting of corpora lutea and large antral, preovulatory, and atretic cyst-like follicles across sections. Seven representative sections per ovary, at least 300 µm apart, were analyzed to count the total number of preantral and small antral follicles using a light microscope (Axio Imager A1; Carl Zeiss, Oberkochen, Germany).

### 2.7 Immunohistochemistry (IHC) for FSHR

Immunohistochemical staining of ovarian tissue sample sections was performed using the avidin-biotin immunoperoxidase method and a Histofine SAB-PO kit (Nichirei Biosciences, Tokyo, Japan). Endogenous peroxidase activity and nonspecific IgG binding were blocked by room temperature incubation in 0.3% hydrogen peroxide in methanol for 20 min at 20–25°C and 10% normal goat serum in phosphate-buffered saline for 10 min, respectively. Tissue sections were incubated for 16 h at 4°C with polyclonal rabbit anti-FSHR antibody (bs-0895R; 1:100 dilution; Bioss Antibodies, Woburn, Massachusetts, USA), followed by incubation with a horseradish peroxidase-conjugated secondary antibody for 10 min at 20–25°C. All slides were incubated with 3,3′-diaminobenzidine tetrahydrochloride, counterstained with hematoxylin, dehydrated, and mounted. For semi-quantification, FSHR-positive GCs on each slide were counted using the ImageJ software (https://imagej.nih.gov/ij/) and a modified H-score (18). The score for the FSHR-positive area was calculated by classifying the staining intensity in the GC layer fields on a four-point scale (0 = negative; 1, weak; 2, moderate, and 3 = strong). Representative images of the FSHR-positive area (score: 1+ [weak] and 3+ [strong]) are shown in **Supplementary Figure 1**. The percentage of GCs at each intensity level was calculated in a possible range of 0–300, using the following formula: [1 × (% cells with intensity 1+) + 2 × (% cells with intensity 2+) + 3 × (% cells with intensity 3+)]. Slides were imaged using a BZ-9000 microscope (Keyence Corporation, Osaka, Japan). The results of staining without primary antibody as negative control are shown in **Supplementary Figure 2**.

### 2.8 Preparation and primary culture of GCs from rat ovaries

GCs from rat ovaries were prepared as previously described with slight modifications (19). Briefly, female Wistar rats (3-week-old) and female offspring rats (PND 21) treated prenatally with vehicle or DHT were injected with 10 IU pregnant mare serum gonadotropin (Aska Animal Health, Tokyo, Japan). After 48 h, the ovaries were obtained from the rats after euthanasia by exsanguination under deep isoflurane anesthesia. The adipose tissue and capsule surrounding the ovaries were removed. Primary GCs were isolated using a 27G needle puncture and visualized with a stereomicroscope (Nikon, Tokyo, Japan). After filtration through 100-μm and 40-μm cell strainers (Corning, NY, USA), GCs were seeded on 96-well plates, 6-well plates, or 60-mm dishes (Corning Primaria, Corning) at a density of 4×10^4^ cells/cm^2^. The plates were incubated overnight at 37°C with 5% carbon dioxide in phenol red-free medium (i.e., a mixture of Dulbecco’s modified Eagle’s medium and Ham’s F-12 medium [DMEM/F12; Thermo Fisher Scientific, Waltham, MA, USA]) supplemented with 10% fetal bovine serum (FBS; JRH Biosciences, Lenexa, KS, USA) and antibiotic-antimycotic (100 μg/mL streptomycin and 100 IU/mL penicillin; Thermo Fisher Scientific). After incubation, the medium was replaced with a serum-free medium (i.e., DMEM/F12 supplemented with 0.1% bovine serum albumin [Sigma-Aldrich, Saint Louis, MO, USA] containing 1% non-essential amino acid solution [MP Biomedicals, Irvine, CA, USA], 2.5 μg/mL transferrin [Nacalai Tesque, Kyoto, Japan], 4 ng/mL sodium selenite [Wako, Osaka, Japan], and 10 ng/mL insulin [Sigma-Aldrich]) to prevent luteinization. GCs were cultured for 24 h before experiments. The medium was replaced with serum-free medium (supplemented with 100 nM androstenedione [i.e., a synthetic source of E2] [Tokyo Chemical Industry], in addition to the aforementioned composition). Cultured GCs were treated with UKT (63–500 μg/mL) and FSH (100 mIU/mL; Merck Serono, Genève, Switzerland), alone or in combination, for 48 h.

### 2.9 cDNA preparation from primary cultured GCs or rat ovarian tissues

Total RNA was extracted from GCs or rat ovarian tissues using QIAzol (Qiagen, Venlo, Netherlands), the RNeasy Universal Tissue Kit (Qiagen), or the AllPrep DNA/RNA/Protein Mini Kit (Qiagen), and reverse-transcribed to cDNA using the TaqMan reverse transcription reagent (Applied Biosystems, Waltham, MA, USA) or ReverTra Ace qPCR RT Master Mix (Toyobo, Osaka, Japan).

### 2.10 Real-time polymerase chain reaction (PCR)

mRNA levels were analyzed using real-time PCR with the TaqMan Fast Advanced Master Mix (Applied Biosystems) and the Prism 7900HT Sequence Detection System (Applied Biosystems) and normalized to that of the endogenous control *Gapdh*. All oligonucleotide primers and fluorogenic probe sets were manufactured by Applied Biosystems (*Fshr*, Rn01648507_m1; *Bmp2*, Rn00567818_m1; *Bmp6*, Rn00432095_m1; *Cyp19a1*, Rn00567222_m1; *Hsd17b*, Rn00563388_m1; *Star*, Rn00580695_m1; *Cyp11a1*, Rn00568733_m1; *Hsd3b*, Rn01789220_m1, and *Gapdh*, Rn01775763_g1).

### 2.11 *In Vitro* measurements

The concentration of E2, bone morphogenetic protein (BMP)-2, or BMP-6 in the medium was determined using a 17β-Estradiol high-sensitivity ELISA kit (Enzo Life Sciences, Farmingdale, NY, USA), Quantikine ELISA BMP-2 Immunoassay (R&D systems, Minneapolis, MN, USA) or Rat BMP-6 ELISA Kit (Novus Biologicals, Centennial, CO, USA). The concentration of cyclic adenosine monophosphate (cAMP) or total protein in the cell lysate was determined using a cAMP complete ELISA kit (Enzo Life Sciences) or a DC Protein Assay kit (Bio-Rad, Hercules CA, USA).

### 2.12 Statistical analysis

Statistically significant differences between the two groups were determined using the Student’s *t*-test or Mann–Whitney *U* test; the latter was used when the variables were not normally distributed. One- or two-way analysis of variance with Dunnett’s multiple comparison test or Holm–Sidak’s multiple comparison tests was used to determine the statistical differences between multiple groups. Values were expressed as dot plots. Means or medians were expressed as horizontal bars or bar graphs. A *P*-value less than 0.05 was considered statistically significant. All analyses were performed using the GraphPad Prism software, version 8.1.2 (GraphPad Software, San Diego, CA, USA).

## 3 Results

### 3.1 Estrous cyclicity and ovarian morphology

**Figure 1:**
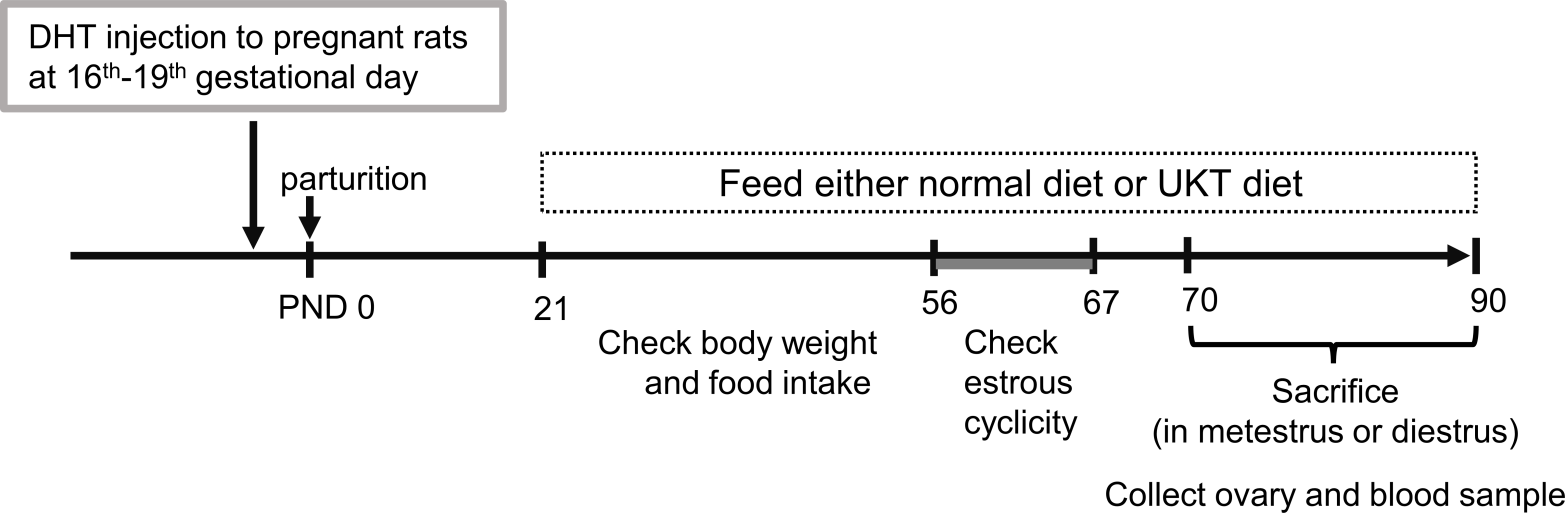
Timeline of the experimental design. PCOS model was generated by prenatal DHT treatment. Female offspring rats fed CE-2 diet (normal diet) or CE-2 diet containing 3% UKT (UKT diet) from PND 21. PCOS, polycystic ovary syndrome; DHT, 5α-dihydrotestosterone; UKT, unkeito; PND, postnatal day.

**Figure 2:**
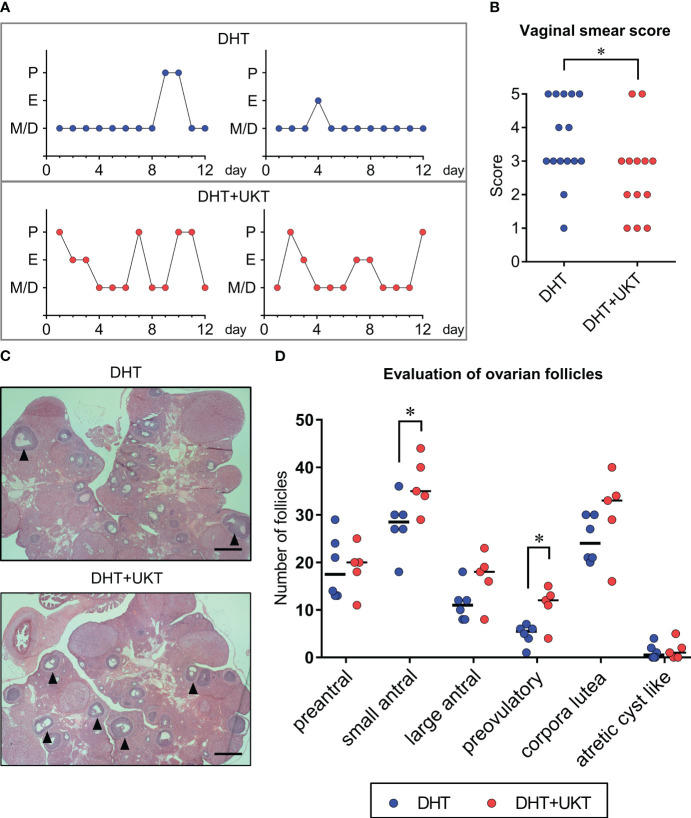
Assessment of estrous cyclicity and ovarian morphology. **(A)** Two representative estrous cycles in each group. **(B)** Quantitative analysis of vaginal smear scores (DHT: n = 15, DHT+UKT: n = 13). **(C)** Representative morphological appearance of affected ovaries from the DHT group (upper panel) or DHT+UKT group (lower panel) (hematoxylin and eosin; scale bars: 500 µm). The arrowhead indicates a preovulatory follicle. **(D)** Evaluation of ovarian follicles in each follicle stage, cyst, or corpus luteum. Horizontal bars indicate medians (DHT: n = 6, DHT+UKT: n = 5). *P < 0.05 (Mann–Whitney *U* test). E, estrus; P, proestrus; M, metestrus; D, diestrus; DHT, 5α-dihydrotestosterone; UKT, unkeito.

The time schedule of the experimental design for drug administration and phenotype evaluation of the rat models is illustrated in **Figure 1**. Although none of the rats in the DHT group had a regular cycle, some rats in the DHT+UKT group showed a better estrous cycle (**Figure 2A**). The smear scores of the DHT+UKT group significantly improved compared to those of the DHT group (**Figure 2B**).

We assessed the follicle count, as described previously (12), and found that the number of small antral and preovulatory follicles in the DHT+UKT group were significantly increased compared to those in the DHT group (**Figures 2C, D**). Although the number of large antral follicles and corpora lutea also tended to increase in the DHT+UKT group, this difference was not statistically significant.

### 3.2 Serum concentrations of sex steroid hormones and gonadotropins

**Figure 3:**
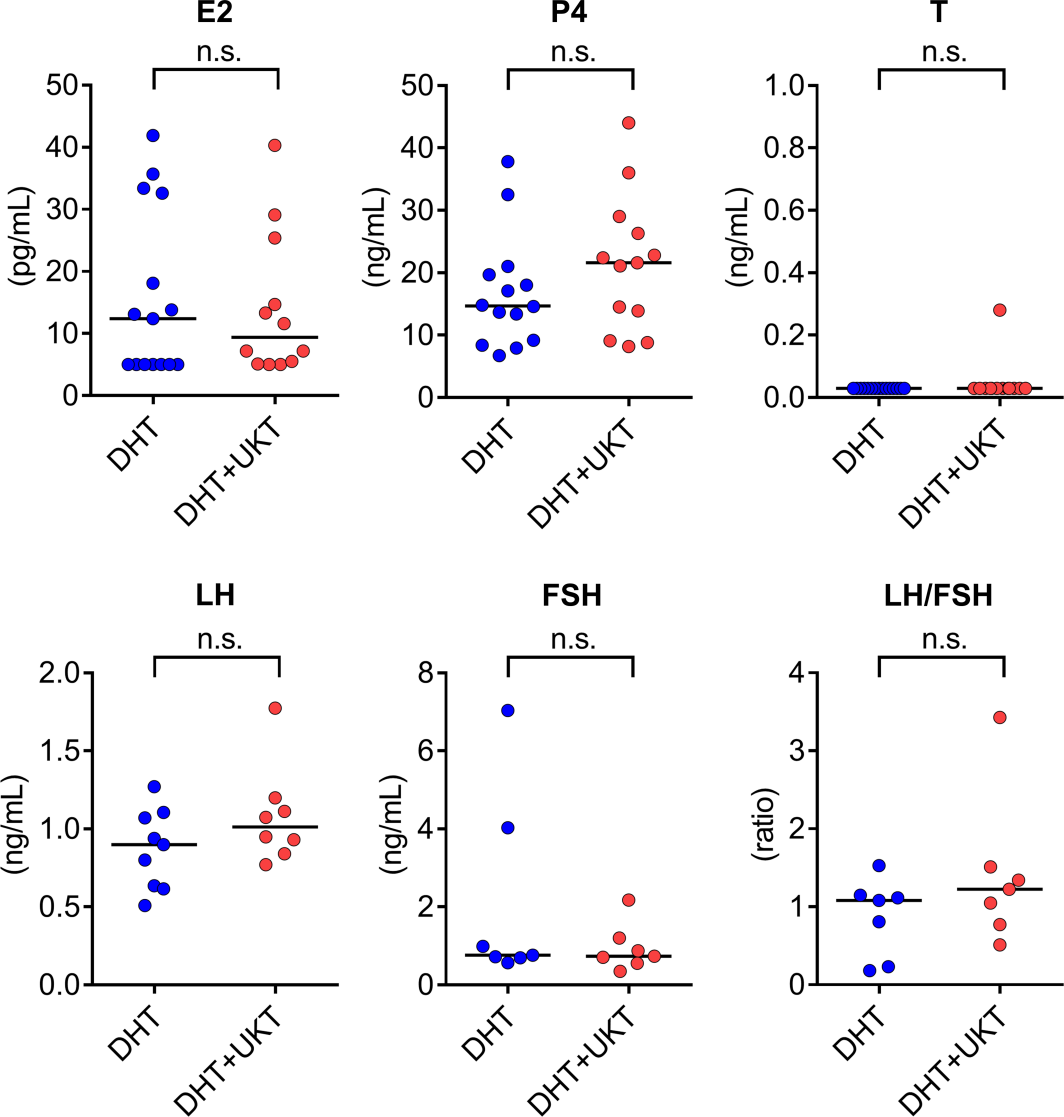
Serum concentrations of sex steroid hormones and gonadotropins. Horizontal bars indicate medians (DHT: n = 7–15, DHT+UKT: n = 7–13). E2, estradiol; P4, progesterone; T, testosterone; LH, luteinizing hormone; FSH, follicle-stimulating hormone; DHT, 5α-dihydrotestosterone; UKT, unkeito; n.s., not significant (Mann–Whitney *U* test).

Serum sex steroid hormone and gonadotropin levels were measured during diestrus and metoestrus. Some samples were below the limit of quantitation for LH and FSH. Serum P4 levels were higher in the DHT+UKT group than in the DHT group, although the difference was not statistically significant. Serum E2, T, LH, FSH, and LH/FSH ratios were similar between the two groups (**Figure 3**).

### 3.3 *Fshr*, *Bmp2*, and *Bmp6* expression levels in ovarian tissues

**Figure 4:**
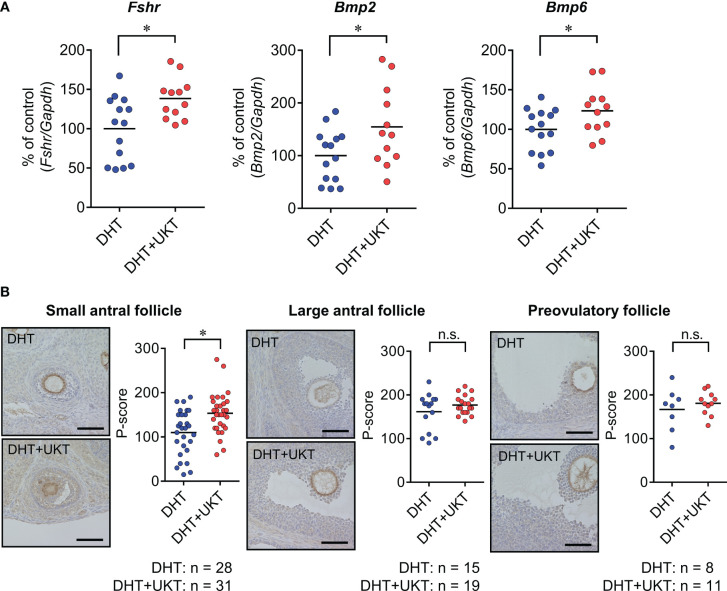
Expressions of *Fshr*, *Bmp2*, and *Bmp6* in ovaries. **(A)** Relative *Fshr, Bmp2*, and *Bmp6* mRNA expression levels in ovaries were quantified using real-time PCR. Expression levels are shown relative to those of *Gapdh*. Horizontal bars indicate means (DHT: n = 14, DHT+UKT: n = 12). **(B)** Immunohistochemical analysis of ovaries from DHT rats (upper panel) or DHT+UKT rats (lower panel) with staining for FSHR. Representative FSHR immunohistochemistry results for ovarian follicles in the small antral, large antral, and preovulatory stages (left panel, scale bars: 40 µm) and quantitative analysis of the P-score in the GC layer (right panel). Horizontal bars indicate means (n is shown in Figure). *P < 0.05 (unpaired *t-*test). *Fshr*, follicle-stimulating hormone receptor; *Bmp*, bone morphogenetic protein; PCR, polymerase chain reaction; *Gapdh*, glyceraldehyde-3-phosphate dehydrogenase. DHT, 5α-dihydrotestosterone; UKT, unkeito; P-score, FSHR-positive area ratio score; GC, granulosa cell; n.s., not significant.

We determined the mRNA expression levels of *Fshr, Bmp2*, and *Bmp6* in the ovaries, which were significantly higher in the DHT+UKT group than in the DHT group (**Figure 4A**). Among the expressions of the other genes involved in follicle development and steroid synthesis, growth and differentiation factor (*Gdf9)*, inhibin subtypes (*Inha, Inhba*, and *Inhbb*)*, Star, Cyp11a1, Cyp19a1*, and *Hsd3b*, showed no significant difference between DHT and DHT+UKT groups (**Supplementary Figure 3**).

Next, we assessed the FSHR protein expression levels in the ovaries using western blot analysis and the GCs of each follicle type by IHC. The DHT+UKT group showed a slight increase in ovarian FSHR expression (**Supplementary Figure 4**). Representative images and FSHR-positive area ratio scores (P-scores) are shown in **Figure 4B**. In the small antral follicles, the P-scores of the DHT+UKT group were higher than those of the DHT group. By contrast, the P-scores for the other follicle types did not differ between the two groups.

### 3.4 Experiments using primary cultured GCs from normal rats

**Figure 5:**
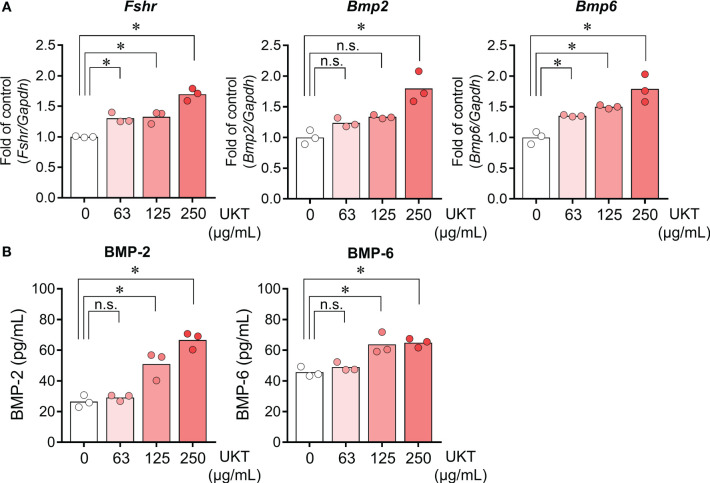
Effect of UKT on the expression of *Fshr* and secretion of BMP-2 and BMP-6 in GCs from normal rats. **(A)** Relative mRNA expression levels of *Fshr*, *Bmp2*, and *Bmp6* mRNA in primary cultured GCs were quantified using real-time PCR. Expression levels are shown relative to those of *Gapdh*. **(B)** Concentrations of BMP-2 and BMP-6 in the culture medium of primary cultured GCs were determined using an ELISA kit. Bar graphs indicate means (n = 3). *P < 0.05 (Dunnett’s test). UKT, unkeito; *Fshr*, follicle-stimulating hormone receptor; *Bmp*, bone morphogenetic protein; GC, granulosa cell; PCR, polymerase chain reaction; *Gapdh*, glyceraldehyde-3-phosphate dehydrogenase; n.s., not significant.

We examined whether UKT affected the expressions of *Fshr*, *Bmp2*, and *Bmp6* in primary cultured GCs derived from normal rats. UKT treatment for 48 h increased expressions of *Fshr*, *Bmp2*, and *Bmp6* in a dose-dependent manner (**Figure 5A**). The levels of BMP-2 and BMP-6 in the culture medium were increased following UKT treatment, indicating stimulation of BMP-2 and BMP-6 secretion (**Figure 5B**).

**Figure 6:**
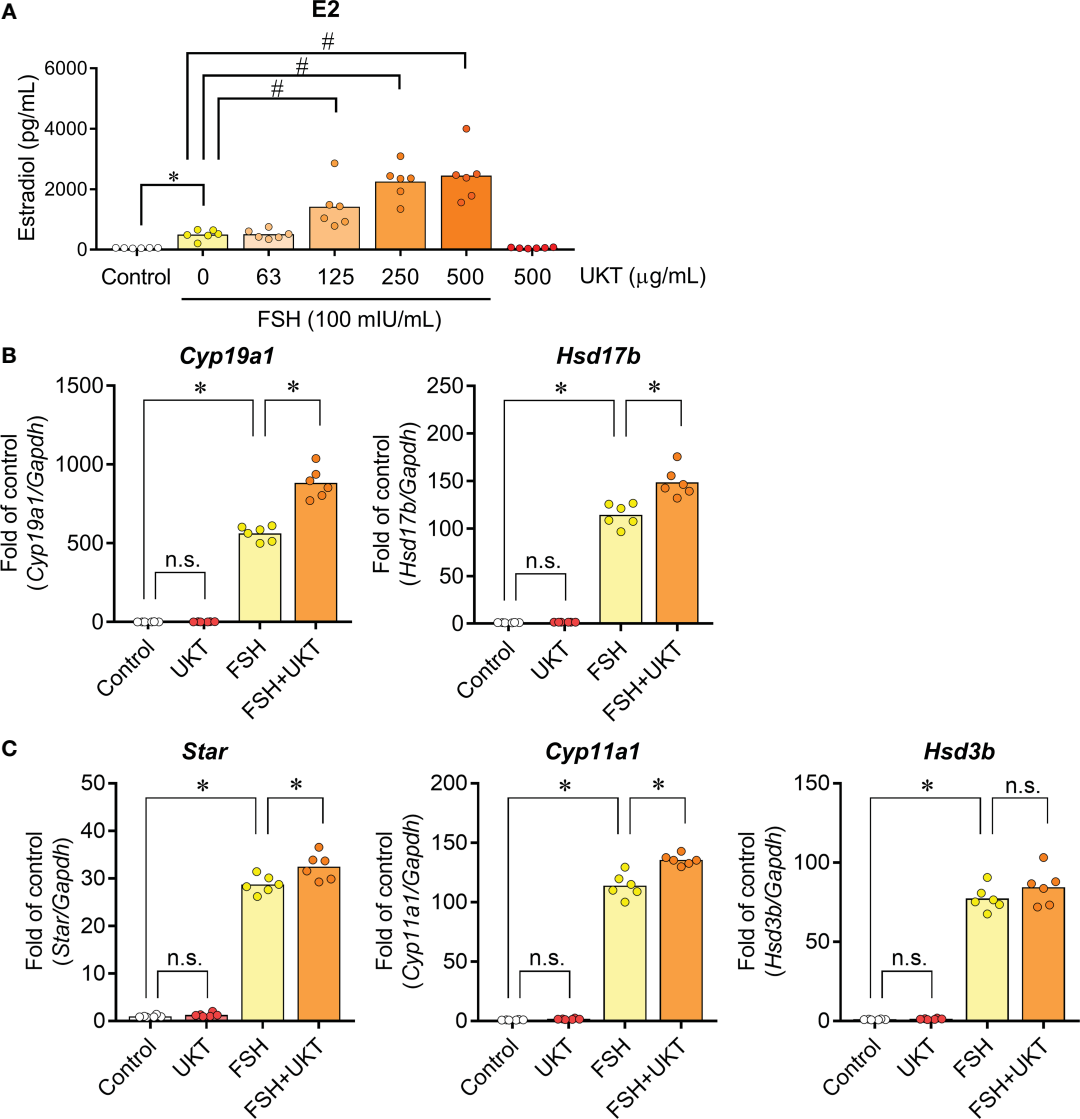
Estradiol production and expression of genes contributing to steroid hormone synthesis in GCs from normal rats. **(A)** Effect of UKT on FSH-induced estradiol production in GCs. The concentration of estradiol in the culture medium was quantified using an ELISA kit. Bar graphs indicate means (n = 6). *P < 0.05 (Mann–Whitney *U* test), #*P <*0.05 (Dunnett’s test). **(B, C)** Relative mRNA expression levels of *Cyp19a1, Hsd17b, Star, Cyp11a1*, and *Hsd3b* were quantified using real-time PCR. Expression levels are shown relative to those of *Gapdh*. Bar graphs indicate means (n = 6). *P < 0.05 (Holm–Sidak’s test). GC, granulosa cell; UKT, unkeito (250 µg/mL); FSH, follicle-stimulating hormone; ELISA, enzyme-linked immunosorbent assay; *Cyp19a1*, cytochrome P450 19A1; *Hsd17b*, 17β-hydroxysteroid dehydrogenase; *Star*, steroidogenic acute regulatory protein; *Cyp11a1*, cytochrome P450 11A1; *Hsd3b*, 3β hydroxysteroid dehydrogenase; PCR, polymerase chain reaction; *Gapdh*, glyceraldehyde-3-phosphate dehydrogenase; n.s., not significant.

We also assessed the effect of FSH responsiveness, as evidenced by FSH-induced E2 production and related gene expression. FSH treatment for 48 h significantly increased E2 production compared to that in the control, whereas UKT enhanced FSH-induced E2 production in a dose-dependent manner (**Figure 6A**). By contrast, UKT treatment alone did not alter E2 production. Similar results were observed for the expression of *Cyp19a1* and *Hsd17b* mRNA (**Figure 6B**). Concerning the genes involved in P4 production, UKT enhanced the levels of *Star* and *Cyp11a1* mRNA but not *Hsd3b* in FSH-treated GCs (**Figure 6C**).

### 3.5 Experiments using primary cultured GCs from DHT-treated rats

**Figure 7:**
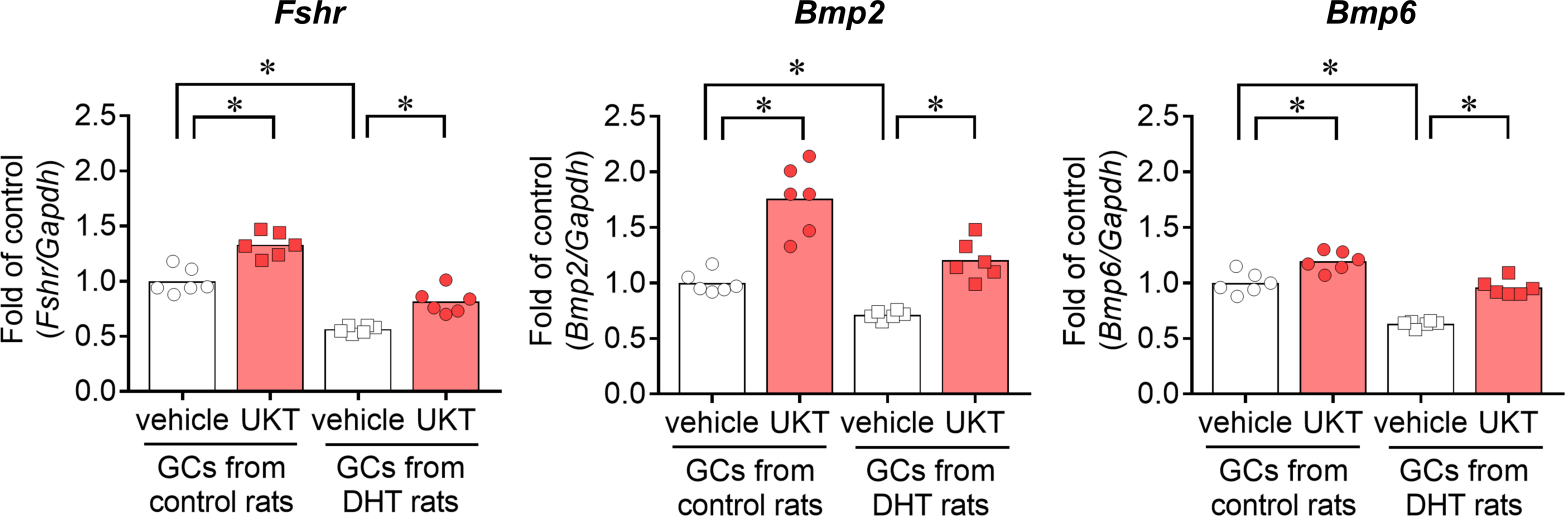
*Fshr*, *Bmp2*, and *Bmp6* expression in GCs from control or DHT-treated rats. Relative mRNA expression levels of *Fshr*, *Bmp2*, and *Bmp6* in GCs were quantified using real-time PCR. Expression levels are shown relative to those of *Gapdh*. Bar graphs indicate means (n = 6). *P < 0.05 (Holm–Sidak’s test). *Fshr*, follicle-stimulating hormone receptor; *Bmp*, bone morphogenetic protein; GC, granulosa cell; DHT, 5α-dihydrotestosterone; UKT, unkeito (250 µg/mL); PCR, polymerase chain reaction; *Gapdh*, glyceraldehyde-3-phosphate dehydrogenase.

**Figure 8:**
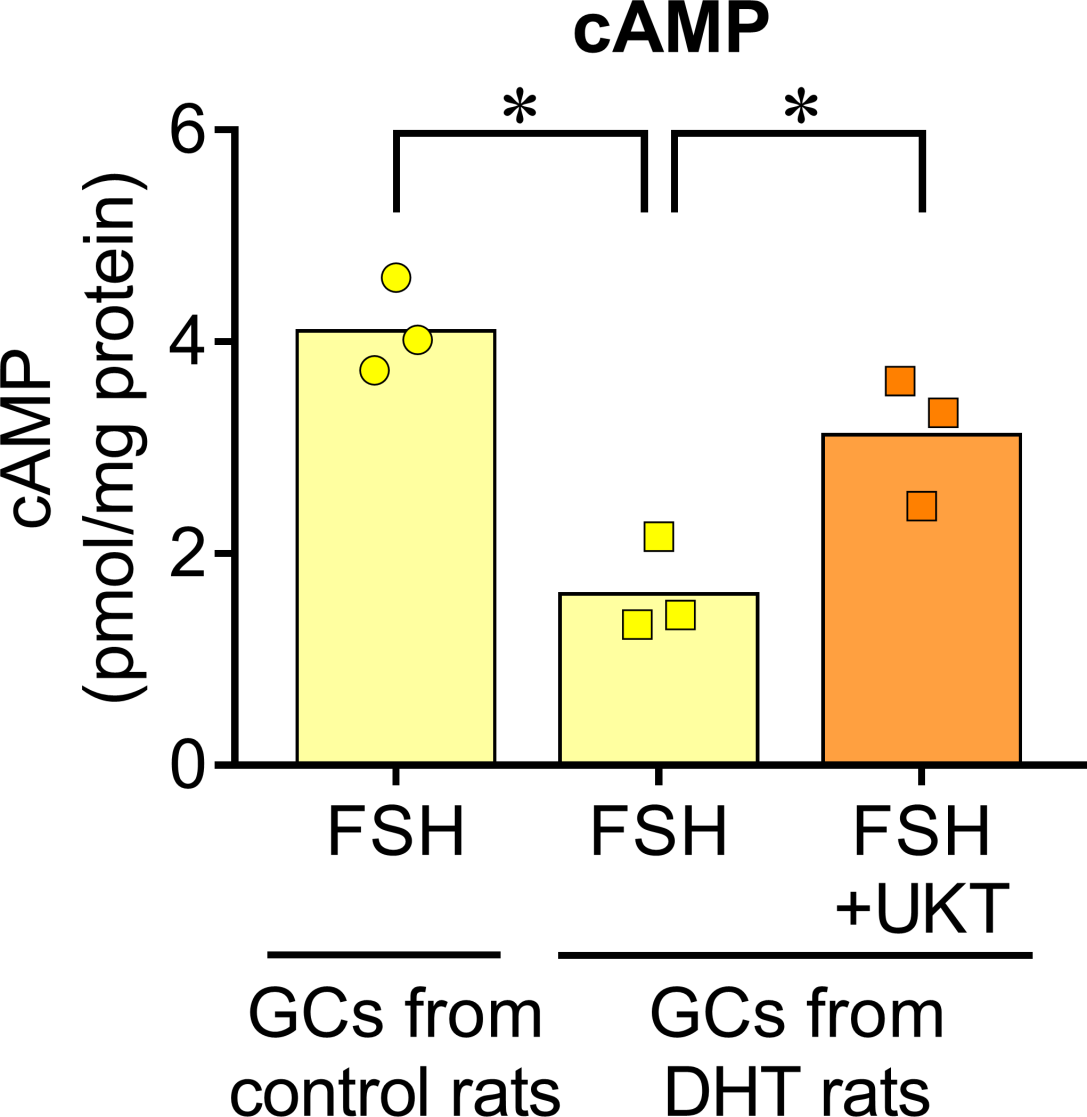
FSH-induced cAMP increase in GCs of control or DHT-treated rats. The cAMP concentration in the GCs lysates was determined using an ELISA kit and corrected for the total protein concentration. Bar graphs indicate means (n = 3). *P < 0.05 (Holm–Sidak’s test). UKT, unkeito (250 µg/mL); FSH, follicle-stimulating hormone; cAMP, cyclic adenosine monophosphate; GC, granulosa cell; DTH, 5α-dihydrotestosterone; ELISA, enzyme-linked immunosorbent assay.

We examined the mRNA expressions of *Fshr*, *Bmp2*, and *Bmp6* in the GCs of control and DHT rats and found them to be lower in the GCs of DHT rats than in those of the control rats. However, UKT upregulated the mRNA expression levels in the GCs of DHT rats (**Figure 7**). UKT also increased the FSHR protein expression in GCs (**Supplementary Figure 5**). FSH-induced cAMP production, an index of FSH responsiveness, was lower in the GCs of DHT rats than in control rats and was restored following UKT treatment in the GCs of DHT rats (**Figure 8**).

**Figure 9:**
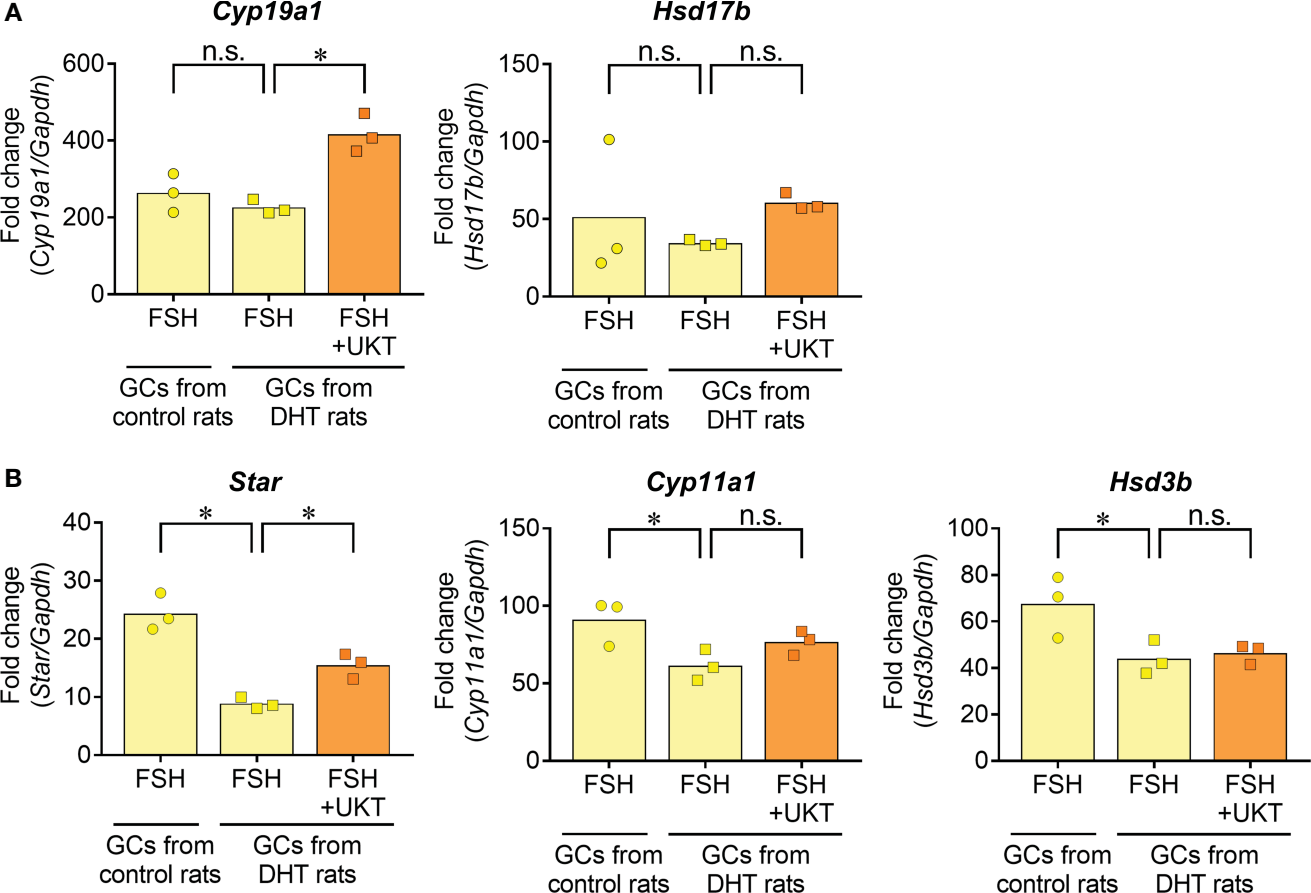
FSH-induced gene expression contributing to steroid hormone synthesis in GCs from control or DHT-treated rats. **(A, B)** Data indicate fold changes in response to FSH in GCs from DHT-treated and vehicle-treated (control) rats. mRNA expression levels of *Cyp19a1, Hsd17b, Star, Cyp11a1* and *Hsd3b* were quantified using real-time PCR. Expression levels are shown relative to those of *Gapdh*. Bar graphs indicate means (n = 3). *P < 0.05 (Holm–Sidak’s test). UKT, unkeito (250 µg/mL); FSH, follicle-stimulating hormone; GC, granulosa cell; DHT, 5α-dihydrotestosterone; *Cyp19a1*, cytochrome P450 19A1; *Hsd17b*, 17β-hydroxysteroid dehydrogenase; *Star*, steroidogenic acute regulatory protein; *Cyp11a1*, cytochrome P450 11A1; *Hsd3b*, 3β hydroxysteroid dehydrogenase; PCR, polymerase chain reaction; *Gapdh*, glyceraldehyde-3-phosphate dehydrogenase; n.s., not significant.

Next, we examined FSH-induced expression of genes involved in sex steroid hormone synthesis (**Figure 9**). No significant differences were observed for *Cyp19a1* and *Hsd17b* in the GCs of DHT and control rats, whereas the expressions of *Star*, *Cyp11a1*, and *Hsd3b* were downregulated in the GCs of DHT rats compared with those of control rats. An enhanced effect of UKT on FSH-induced *Cyp19a1* and *Star* mRNA expression was observed in the GCs of DHT rats.

## 4 Discussion

Our study indicated that UKT increased the number of small antral and preovulatory follicles and improved the irregular estrous cycle in a rat model of PCOS, both associated with enhanced FSHR protein expression through the upregulation of *Bmp2* and *Bmp6* expression. Moreover, UKT treatment enhanced FSH responsiveness in GCs cultured from PCOS rats. These findings suggest that UKT stimulates follicular development by potentiating FSHR function in a rat model of PCOS.

Researchers have attempted to establish several animal models with distinct phenotypes to elucidate the pathogenesis of PCOS. Prenatal androgen (PNA) exposure induces PCOS-like phenotypes with an increase in plasma testosterone, LH pulse frequency, and the ratio of LH and FSH postnatally in the female offspring. A greater number of preantral and small follicles and fewer preovulatory follicles and corpus luteum are observed following PNA treatment (13, 20–22). In agreement with these reports, we also previously confirmed that prenatally DHT-exposed rats exhibit irregular estrous cycles with PCOS-like ovarian morphology, high LH levels, and normal body weight. Follicle count evaluation showed that the ovaries of prenatally DHT-treated rats had fewer growing preovulatory follicles and fewer corpus luteum than vehicle-treated rats (12). Therefore, our rat model could mimic non-obese PCOS patients, observed more often in the Asian ethnicity (23, 24). In the present study, UKT reduced estrous cycle abnormalities in rats with PCOS. This observation is consistent with a previous clinical report (2). Small antral and preovulatory follicle numbers in PCOS rats were increased by UKT treatment, suggesting that UKT could promote follicle development in PCOS.

Follicle development is controlled by gonadotropin-dependent and -independent mechanisms at each stage (25–27). Responsiveness to FSH is a key factor in follicular development and functions through the gonadotropin-dependent pathway. In a gonadotropin-independent system, local paracrine and autocrine factors are deeply involved, and their crosstalk regulates follicle development (28). In this study, there were no significant changes in the serum levels of FSH, LH, and sex steroid hormones during diestrus and metestrus between the UKT-treated and control groups, although serum progesterone levels tended to be higher in PCOS rats treated with UKT than in control rats. These findings suggest that UKT has little effect on gonadotropin secretion in rats with PCOS.

Recently, Owens et al. (10) reported that *FSHR* expression is significantly decreased in the GCs of patients with PCOS. We also observed that *Fshr* mRNA expression decreased in GCs from PCOS rats compared to those from normal rats. Therefore, we evaluated the effects of UKT on FSHR expression in rats with PCOS. First, we quantified protein levels using western blotting. FSHR protein expression in the ovaries was higher in the DHT+UKT group than in the DHT group. Immunohistological studies showed that FSHR staining in the GCs of the small antral follicles of the DHT+UKT group was stronger than that in the DHT group, whereas this change was not observed in large or preovulatory follicles. These results were supported by the quantification of ovarian *Fshr* mRNA expression and increased FSHR protein expression in the GCs. FSH stimulates ovarian follicle growth, and small antral follicles are sensitive to FSH (8). Accordingly, the promoting effect of UKT on the development of small antral follicles in rats with PCOS could be mediated by the activation of FSHR expression, which might affect the increase in large antral and preovulatory follicles.

The transforming growth factor beta superfamily members, such as bone morphogenetic proteins (BMPs), growth and differentiation factor (GDF)-9, and inhibin, play a central role in normal follicle growth (29). In this study, we observed that the levels of *Bmp2* and *Bmp6* mRNAs but not those of GDF-9 and inhibin subtypes, increased in the ovaries in the DHT+UKT group. BMPs derived from oocytes and GCs are involved in follicular development through both gonadotropin-dependent and -independent pathways (28). Notably, BMP-2 and BMP-6 stimulate *FSHR* expression in GCs and BMP-6 directly activates GC proliferation (30–32). By contrast, BMP-6 inhibits FSH responsiveness by suppressing adenylyl cyclase activity (33). These conflicting findings may result from differences in the stage of follicle development; however, BMP-6 regulates follicle selection and ovulation rate by modulating follicle sensitivity to FSH (34). Cell culture experiments revealed that *Bmp2* and *Bmp6* mRNA expressions were significantly lower in the ovarian GCs of PCOS rats than in normal rats, whereas UKT treatment increased BMP secretion, accompanied by the upregulation in *Fshr* and *Bmps* expressions in primary cultured GCs from PCOS rats. Collectively, our findings suggest that UKT elevates FSHR expression through autocrine and paracrine actions by enhancing *Bmp2* and *Bmp6* expression levels.

Gonadotropin stimulates steroid hormone production associated with ovarian follicle growth (35). FSH induces the expression of CYP19A1 and HSD17B, which are involved in E2 synthesis from androstenedione in GCs (36, 37). In this study, an increase in E2 secretion was observed in primary GCs cultured with FSH. In these cultured GCs, UKT potentiated E2 production, accompanied by increased *Cyp19a1* and *Hsd17b* expression, suggesting enhanced FSH responsiveness. Additionally, FSH-induced intracellular cAMP levels in ovarian GCs were lower in PCOS rats than in normal rats, which showed recovery after UKT treatment. Therefore, these observations suggest that UKT can improve decreased FSHR signaling in PCOS, which is related to increased *Cyp19a1* expression. By contrast, the expressions of STAR, CYP11A1, and HSD3B, which are involved in progesterone production, are elevated in cultured GCs in the presence of FSH (38). In the present study, UKT partially potentiated these enzymes in FSH-stimulated GCs. UKT reportedly increases progesterone levels in the mid-luteal phase in patients with luteal phase defects (3). These findings suggest that UKT may contribute to an overall increase in ovulation frequency and improve estrous cycles in patients with PCOS.

No significant effect of UKT on the mRNA expression of these steroid hormone-producing enzymes was observed in the ovaries of the PCOS rats. The difference in findings between the *in vivo* and *in vitro* studies may possibly be due to the heterogeneity of ovarian follicles in rats with PCOS because UKT increased FSHR expression in only small antral follicles but not in other follicles. Furthermore, UKT had little effect on serum steroid hormone levels, suggesting the participation of autocrine and paracrine actions of steroid hormones in the ovarian follicles of PCOS rats; therefore, there may be no significant change in systemic steroid hormones.

Three-dimensional high-performance liquid chromatography revealed that UKT contained several constituents (**Supplementary Figure 6**). Glycyrrhizin derived from Glycyrrhiza roots improves fertility as evidenced in an *in vitro* study (39). Therefore, glycyrrhizin may be an active constituent involved in follicular development.

However, this study had some limitations. PCOS is a complex endocrine disorder associated with elevated LH, anti-Mullerian hormone, androgens, and insulin resistance. Among them, LH and its receptor are closely associated with follicle development and the pathogenesis of PCOS (1); however, it is unknown how LH is related to FSH responsiveness during follicle development in PCOS and its effects on UKT treatment. Further studies are needed to address this question.

In conclusion, our findings revealed that UKT decreased estrous cycle abnormalities and increased follicular development in PCOS rat models by potentiating FSH responsiveness, which was mediated by the upregulation of BMP-2 and BMP-6 expressions. Therefore, restoring the FSHR dysfunction in the small antral follicles could alleviate the PCOS phenotype. UKT may effectively improve ovarian dysfunction in patients with PCOS.

## Data availability statement

The original contributions presented in the study are included in the article/**Supplementary Material**. Further inquiries can be directed to the corresponding author.

## Ethics statement

The animal study was approved by the Animal Experiment Committee of Nagoya University Graduate School of Medicine (approval no. M210146) and the Animal Experiment Committee of Tsumura & Co. (approval nos. 19-052, 19-059, and 20-035). The study was conducted in accordance with the local legislation and institutional requirements.

## Author contributions

SO conceptualized and designed the study. SY and TS performed animal experiments. TS performed the cell culture experiments. B, SO, NM, AM, and NN provided technical and material support. SY, TS, and NF drafted the manuscript. SO supervised the study and edited the manuscript. TN, MG, HK, and CM supervised the study. All authors contributed to the article and approved the submitted version.
