# Supplementary material for: Unkeito promotes follicle development by restoring reduced follicle-stimulating hormone responsiveness in rats with polycystic ovary syndrome
Source: Front Endocrinol (Lausanne). 2023 Sep 18;14:1228088. doi: 10.3389/fendo.2023.1228088 (PMC10545092; doi:10.3389/fendo.2023.1228088)
Supplement: Supplementary file 1 [file DataSheet_1.docx]

Supplementary Material

Unkeito Promotes Follicle Development by Restoring Reduced Follicle-Stimulating Hormone Responsiveness in Rats with Polycystic Ovary Syndrome

Sayako Yoshita, Satoko Osuka*, Tomofumi Shimizu, Naoki Fujitsuka, Chinami Matsumoto, Bayasula, Natsuki Miyake, Ayako Muraoka, Natsuki Nakanishi, Tomoko Nakamura, Maki Goto, Hiroaki Kajiyama

*** Correspondence:** Satoko Osuka, MD, PhD

satokoosuka@med.nagoya-u.ac.jp

# Supplementary Materials and Methods

## Oligonucleotide primers used in TaqMan RT-PCR experiment

All oligonucleotide primers and fluorogenic probe sets for TaqMan RT-PCR experiment were manufactured by Applied Biosystems (*Gdf9*: Rn00572328_m1; *Inha*: Rn00561423_m1; *Inhba*: Rn01538592_m1; *Inhbb*: Rn01753772_m1; *Star*: Rn00580695_m1; *Cyp11a1*: Rn00568733_m1; *Cyp19a1*: Rn00567222_m1; *Hsd3b*: Rn01789220_m1 and *Gapdh*: Rn01775763_g1).

## Three-dimensional high-performance liquid chromatography (HPLC)

The HPLC apparatus comprised analysis system software (Shimadzu LC 10A [CLASS-M10A, version 1.64], Shimadzu Corporation, Kyoto, Japan) equipped with a multiple-wavelength detector (ultraviolet 200–400 nm; Shimadzu SPD-M10AVP, diode array detector) and an auto-injector (Shimadzu CTO-10AC). HPLC conditions were as follows: column, octadecyl silyl silica gel column (TSK-GEL 80TS, 250×4.6 mm i.d.; TOSOH, Tokyo, Japan); temperature, 40°C; flow rate, 1.0 mL/min; and eluent, (A) 0.05 M AcONH_4_ (pH 3.6), and (B) 100% CH_3_CN. A linear gradient of 90% A and 10% B was used along with a linear gradient of 0% A and 100% B for 60 min, followed by 100% B for another 20 min.

One granule of UKT (1.0 g) was extracted with methanol (20 mL) using ultrasonication for 30 min and centrifugation at 3,000 rpm for 5 min. The supernatant was filtered through a membrane filter (0.45 μm) and subsequently subjected to HPLC analysis (30 μL).

Standard compounds isolated, purified, and identified using mass spectrometry, infrared spectrometry, and nuclear magnetic resonance spectroscopy from botanical raw materials in UKT were analyzed under the same conditions. Data from the ultraviolet spectrum and column retention time were used to create a chromatogram library. The degree of coincidence and homogeneity of the peaks was evaluated using the peak detector of the library (an auxiliary function of HPLC).

## Supplementary Figures


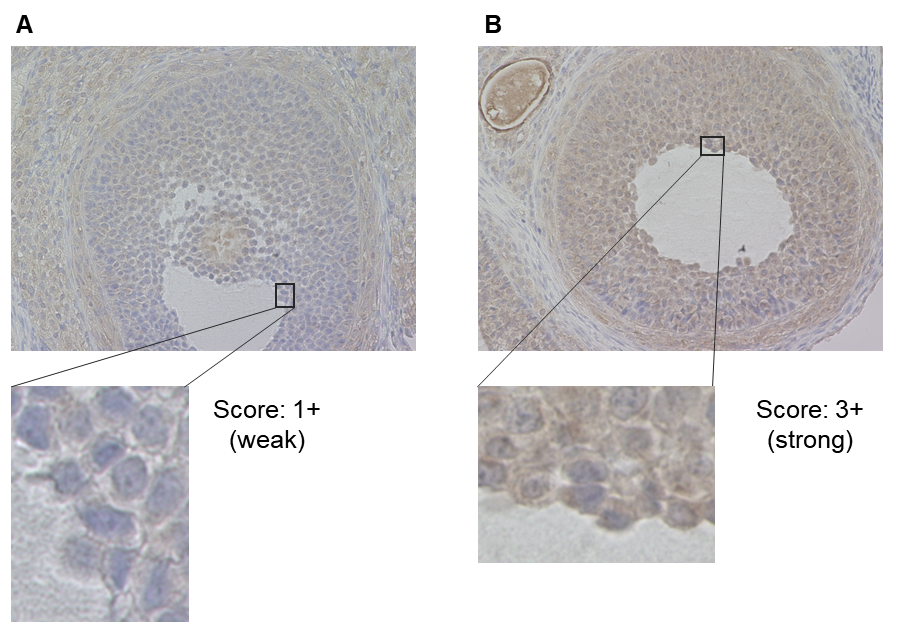


**Supplementary Figure 1.** Representative images of FSHR-positive area (score: 1+ [A, weak] and 3+ [B, strong]) in the GC layer fields in immunohistochemistry for FSHR.


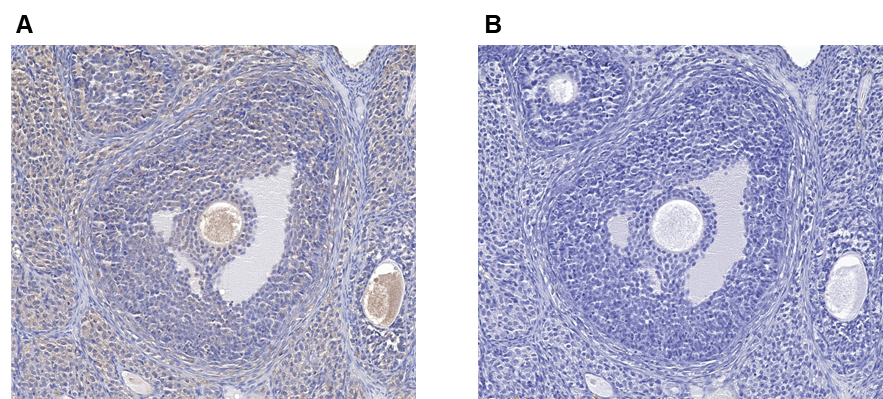


**Supplementary Figure 2**. Immunohistochemistry for FSHR with primary antibody (A) and without primary antibody (B, negative control).


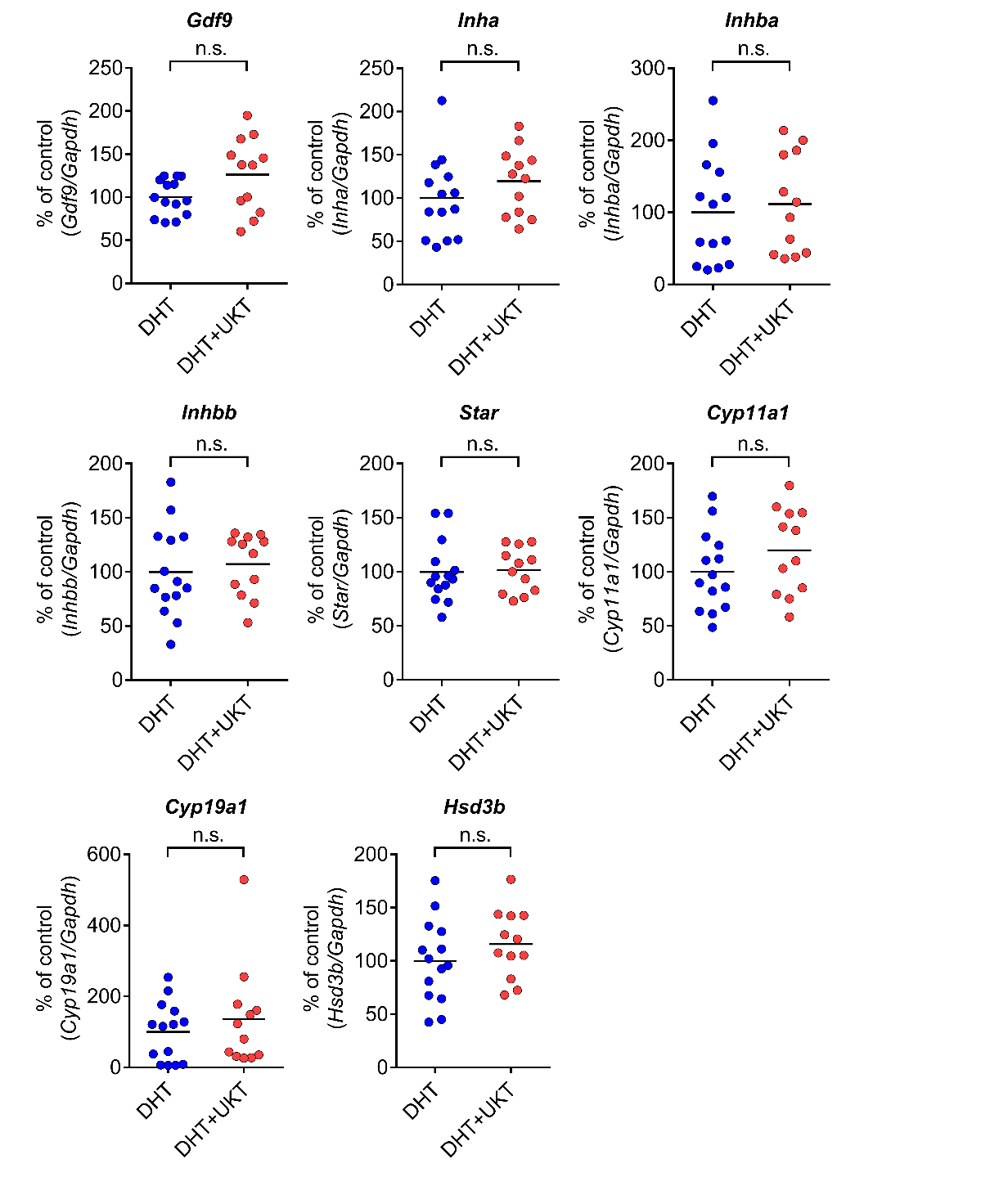


**Supplementary Figure 3.** Relative mRNA expression levels of *Gdf9, Inha, Inhba, Inhbb,* *Star, Cyp11a1, Cyp19a1,* and *Hsd3b* in the ovaries were quantified using real-time PCR. Expression levels are shown relative to those of *Gapdh*. Horizontal bars indicate means (DHT: n = 14, DHT+UKT: n = 12). There were no significant differences between the DHT and DHT+UKT groups (unpaired *t-test*). *Gdf9*, growth differentiation factor 9; *Inha*, inhibin alpha chain; *Inhba*, inhibin beta A chain; *Inhbb*, inhibin beta B chain; *Star*, steroidogenic acute regulatory protein; *Cyp11a1*, cytochrome P450 11A1; *Cyp19a1*, cytochrome P450 19A1; *Hsd3b*, 3β hydroxysteroid dehydrogenase; DHT, 5α-dihydrotestosterone; UKT, unkeito; PCR, polymerase chain reaction; *Gapdh*, glyceraldehyde-3-phosphate dehydrogenase. n.s., not significant.


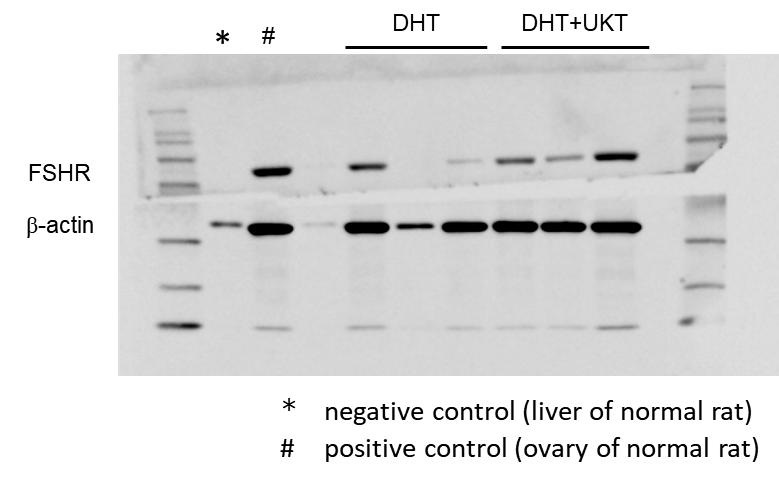


**Supplementary Figure 4.** Western blotting for FSHR and β-actin levels in ovaries from the DHT and DHT+UKT groups.


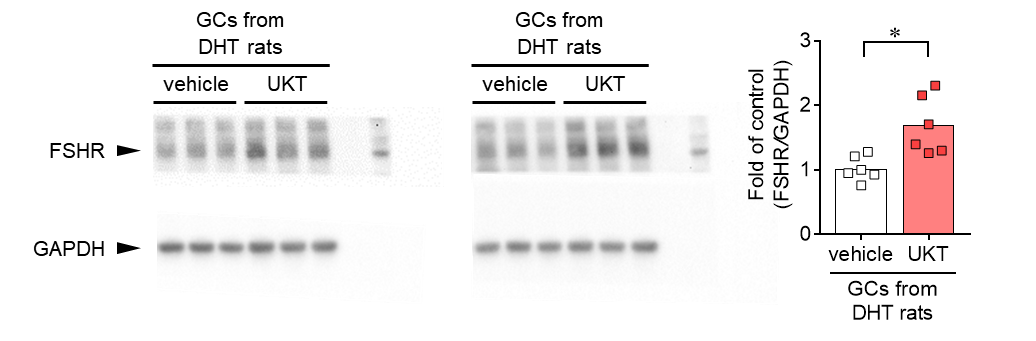


**Supplementary Figure 5.** Relative FSHR expression quantified using western blotting. Bar graphs indicate means (n = 6). **P* < 0.05 (unpaired *t-test*). DHT, 5α-dihydrotestosterone; UKT, unkeito; FSHR, follicle-stimulating hormone receptor; GC, granulosa cell; *Gapdh*, glyceraldehyde-3-phosphate dehydrogenase.


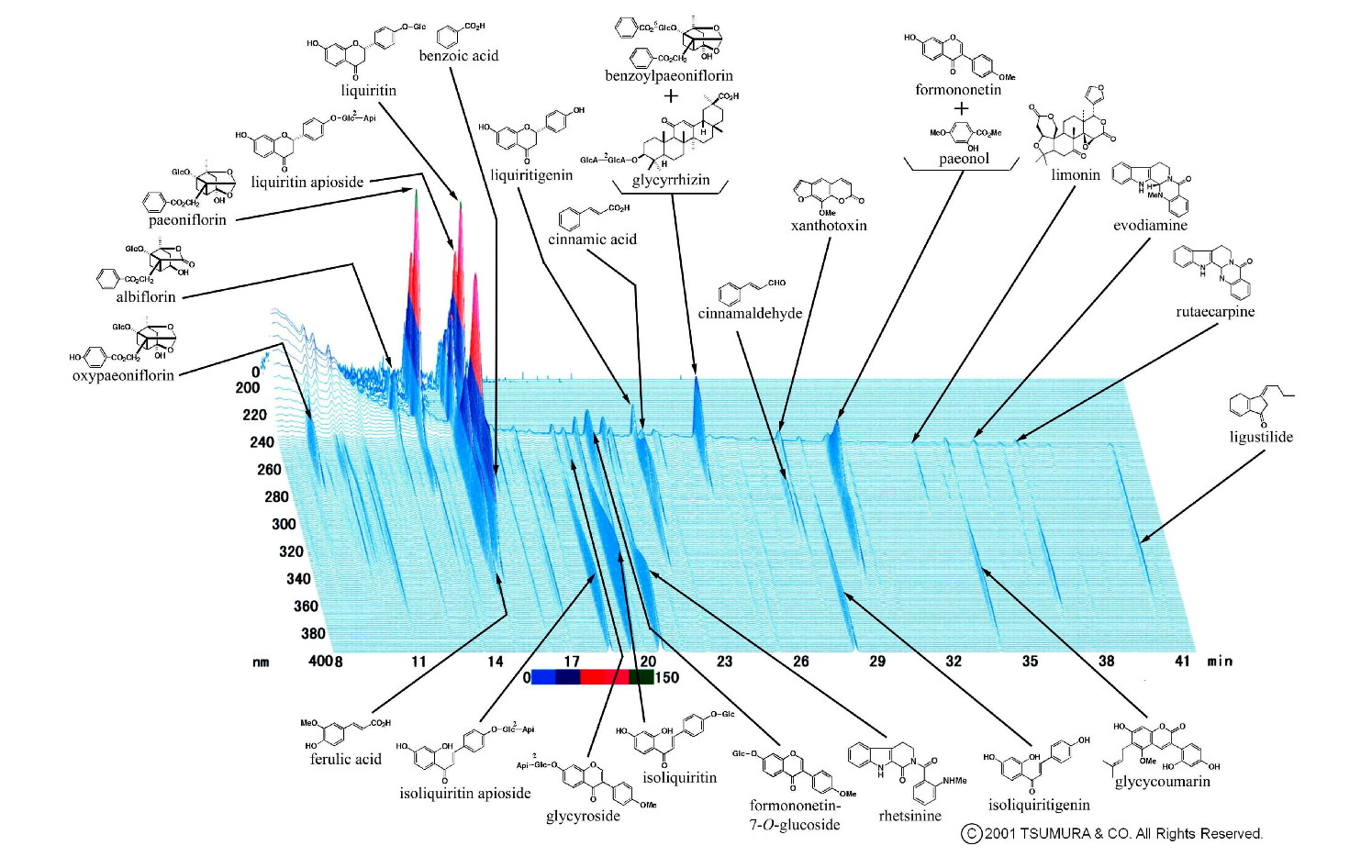


**Supplementary Figure 6.** Three-dimensional high-performance liquid chromatography profile of UKT.
